# Supplementary material for: Interaction effects of significant risk factors on low bone mineral density in ankylosing spondylitis
Source: PeerJ. 2023 Nov 22;11:e16448. doi: 10.7717/peerj.16448 (PMC10676083; doi:10.7717/peerj.16448)
Supplement: Supplemental Information 1 — BMD, bone mineral density; BMI,bodybossindex; TNF, tumour necrosis factor; cDMARDs, conventional DMARDs; BASDAI, Bath Ankylosing Spondylitis Disease Activity Index; BASMI, Bath Ankylosing Spondylitis Metrology Index; BASFI, Bath Ankylosing Spondylitis Functional Index; PGA, patient global assessment; HLA-B27, human leucocyte antigen B27; mSASSS, modified ankylosing spondylitis score; Sacroiliitis average, means average radiological grade of the sacroiliac joint. [file peerj-11-16448-s001.docx]

**Table S1:**

**Baseline characteristics and disease-related variables of the validation set.**

| **Variables** | **All**  **(N=140)** | **Normal BMD**  **(N=63)** | **Low BMD**  **(N=77)** | ***P* value** |
| --- | --- | --- | --- | --- |
| **Demographic variables** |  |  |  |  |
| Male | 105 ( 75.0% ) | 46( 73.0% ） | 59 (76.6%) | 0.624 |
| Age, years | 34.3 (7.5) | 34.1(7.6) | 34.3 (7.6) | 0.455 |
| BMI, kg/m^2^ | 27.3(7.0) | 26.5 (7.1) | 28.0 (7.0) | 0.110 |
| Sport | 40( 28.6%) | 17 (27.0 %) | 23 (29.9 %) | 0.707 |
| Family history | 77(55.0%) | 18(28.6%) | 17(22.1%) | 0.377 |
| Diagnosis duration, years | 6.1(5.0) | 6.2 (7.1) | 6.1 (6.8) | 0.304 |
| Symptoms duration, years | 7.7 (7.6) | 7.5 (7.5) | 7.7 (7.8) | 0.422 |
| Onset age, years | 23.9 (7.8) | 22.9 (7.6) | 24.7 (7.9) | 0.089 |
| Smoking index | 30.7 (79.9) | 30.3 (86.1) | 31.1 (75.0) | 0.477 |
| Smoke duration, years | 3.6(7.2) | 4.4(8.5) | 2.9 (5.8) | 0.105 |
| Cigarettes per day | 3.5(7.6) | 3.6 (7.5) | 3.4 (7.7) | 0.575 |
| Ever smoking | 28 (20.0%) | 10 (15.9%) | 18 (23.4%) | 0.269 |
| Current smoking | 26 (18.6%) | 10(15.9%) | 16 (20.8%) | 0.458 |
| Alcohol duration | 2.4(5.1) | 3.5 (6.7) | 1.6 (3.1) | 0.014 |
| Alcohol history | 45(32.1%) | 22(34.9%) | 23(29.9%) | 0.524 |
| Daily alcohol | 19(13.6%) | 8 (12.7%) | 11 (14.3%) | 0.785 |
| Alcohol frequency | 0.3 (0.5) | 0.3 (0.5) | 0.2(0.5) | 0.077 |
| **Current medication status** |  |  |  |  |
| Patients on TNF inhibitor | 5 (3.6%) | 3 (4.8%) | 2 (2.6%) | 0.492 |
| Patients on NSAIDs | 76(54.3%) | 37 (58.7%) | 39 (50.6%) | 0.340 |
| Patients on cDMARDs | 18(12.9%) | 8(12.7%) | 10(13.0%) | 0.960 |
| **Disease-related variables** |  |  |  |  |
| BASDAI, score | 3.5(1.8) | 3.5 (1.8) | 3.5 (1.9) | 0.477 |
| BASFI, score | 2.4 (2.3) | 2.6 (2.2) | 2.2 (2.3) | 0.128 |
| BASMI, score | 2.2 (2.1) | 1.8 (2.2) | 2.5 (1.9) | 0.023 |
| Night pain, score | 3.6 (1.5) | 3.4 (1.5) | 3.7 (1.5) | 0.192 |
| PGA, score | 3.4 (1.4) | 3.2 (1.5) | 3.5 (1.3) | 0.071 |
| Chest expansion, cm | 3.7 (1.8) | 3.8（1.9） | 3.5 (1.7) | 0.139 |
| modified-Schober, score | 4.1 (1.9) | 4.2 (1.9) | 4.1 (1.9) | 0.413 |
| HLA-B27 Positive | 128 (91.4%) | 57 (90.4%) | 71 (92.2%） | 0.716 |
| Sacroiliitis average, score | 3.2 (0.8) | 2.9 (0.8) | 3.3 (0.7) | 0.001 |
| mSASSS, score | 21.4 (16.2) | 18.6 (15.8) | 23.8 (16.2) | 0.030 |
| Hip involvement | 45(32.1%) | 12 (19.0%) | 33 (42.9%) | 0.003 |
| ESR, mm/h | 19.4 (22.5) | 22.9 (26.1) | 16.6 (18.8) | 0.050 |
| CRP, mg/L | 2.0 (2.7) | 2.3 (2.9) | 1.8 (2.4) | 0.105 |
| ASDAS-CRP, scores | 1.5 (0.7) | 1.4 (0.7) | 1.5 (0.7) | 0.256 |
| **BMD** |  |  |  |  |
| Lumbar spine | 1.1 (0.2) | 1.2 (0.2) | 1.0 (0.2) | 0.002 |
| Femoral neck | 0.9 (0.2) | 1.0 (0.2) | 0.8 (0.1) | <0.001 |
| Total hip | 0.9 (0.3) | 1.1(0.3) | 0.8 (0.1) | <0.001 |

BMD, bone mineral density; BMI, body boss index; TNF, tumour necrosis factor; cDMARDs, conventional DMARDs; BASDAI, Bath Ankylosing Spondylitis Disease Activity Index; BASMI, Bath Ankylosing Spondylitis Metrology Index; BASFI, Bath Ankylosing Spondylitis Functional Index; PGA, patient global assessment; HLA-B27, human leucocyte antigen B27; mSASSS, modified ankylosing spondylitis score; Sacroiliitis average, means average radiological grade of the sacroiliac joint.
