# Supplementary material for: Interaction effects of significant risk factors on low bone mineral density in ankylosing spondylitis
Source: PeerJ. 2023 Nov 22;11:e16448. doi: 10.7717/peerj.16448 (PMC10676083; doi:10.7717/peerj.16448)
Supplement: Supplemental Information 2 — BMD, bone mineral density. [file peerj-11-16448-s002.docx]

**Table S2:**

**Prevalence of low BMD in different sites.**

| **Measurement sites** | **Osteopenia**  **n (%)** | **Osteoporosis**  **n (%)** | **Low BMD**  **n (%)** | **Normal BMD**  **n (%)** |
| --- | --- | --- | --- | --- |
| **All** |  |  |  |  |
| lumber spine | 61 (24.5) | 12 (4.8) | 73 (29.3) | 176 (70.7) |
| femoral neck | 58 (23.3) | 8 (3.2) | 66 (26.5) | 183 (73.5) |
| Total hip | 56 (22.5) | 6 (2.4) | 62 (24.9) | 187 (75.1) |
| **Hip involvement** |  |  |  |  |
| lumber spine | 4 (13.8) | 6 (20.7) | 10 (34.5) | 19 (65.5) |
| femoral neck | 3 (10.3) | 5 (16.3) | 8 .(27.6) | 21 (72.4) |
| Total hip | 3 (10.3) | 8 (27.6) | 11 (37.9) | 18 (62.1) |
| **Without hip involvement** |  |  |  |  |
| lumber spine | 1 (1.7) | 9 (15.0) | 10 (16.7) | 50 (83.3) |
| femoral neck | 0 (0.0) | 9 (15.0) | 9 (15.0) | 51 (85.0) |
| Total hip | 0 (0.0) | 7 (11.7) | 7 (11.7) | 53 (88.3) |

BMD, bone mineral density.
