# Supplementary material for: Interaction effects of significant risk factors on low bone mineral density in ankylosing spondylitis
Source: PeerJ. 2023 Nov 22;11:e16448. doi: 10.7717/peerj.16448 (PMC10676083; doi:10.7717/peerj.16448)
Supplement: Supplemental Information 3 — BMD, bone mineral density. AP, anteroposterior position. [file peerj-11-16448-s003.docx]

**Table S3:**

**Spearman’s correlation between BMD and mSASSS scores in AS patients with syndesmophytes.**

| **BMD** | **mSASSS scores** | |
| --- | --- | --- |
|  | ***r_s_*** | ***P*** |
| AP lumbar spine | 0.201 | 0.024 |
| Femoral neck | -0.156 | 0.081 |
| Total hip | -0.146 | 0.102 |

BMD, bone mineral density. AP, anteroposterior position.
