# Supplementary material for: Interaction effects of significant risk factors on low bone mineral density in ankylosing spondylitis
Source: PeerJ. 2023 Nov 22;11:e16448. doi: 10.7717/peerj.16448 (PMC10676083; doi:10.7717/peerj.16448)
Supplement: Supplemental Information 4 — BASMI, Bath Ankylosing Spondylitis Metrology Index; BASFI, Bath Ankylosing Spondylitis Functional Index; BMI, body boss index; PGA, patient global assessment. FDR, False Discovery Rate. OR, odds ratio; 95% CI, 95% confidence interval. Sacroiliitis average, means average radiological grade of the sacroiliac joint. [file peerj-11-16448-s004.docx]

**Table S4：**

**The results of univariate logistic regression analysis were corrected by FDR using BH method**

| **Variables** | **Femoral neck** | | | | | | | |  | |  | **Total hip** | | |  |  |
| --- | --- | --- | --- | --- | --- | --- | --- | --- | --- | --- | --- | --- | --- | --- | --- | --- |
|  | **OR** | **95% CI** | ***P*** | **FDR- *P*** | | |  | **OR** | | **95% CI** | | | ***P*** | **FDR- *P*** | | |
| **Unadjusted** |  |  |  | | |  |  |  | |  | | |  |  | | |
| Chest expansion | 0.81 | 0.70 to 0.94 | 0.005 | | 0.017 | |  | 0.86 | | 0.74 to 0.99 | | | 0.038 | 0.056 | | |
| BASMI | 1.21 | 1.05 to 1.39 | 0.007 | | 0.015 | |  | 1.17 | | 1.01 to 1.34 | | | 0.032 | 0.056 | | |
| BASFI | 1.04 | 0.88 to 1.24 | 0.640 | | 0.662 | |  | 1.15 | | 0.98 to 1.36 | | | 0.095 | 0.095 | | |
| Total mSASSS | 1.02 | 1.01 to 1.03 | 0.003 | | 0.011 | |  | 1.02 | | 1.00 to 1.03 | | | 0.011 | 0.030 | | |
| ASDAS-CRP | 1.22 | 0.84 to 1.78 | 0.286 | | 0.393 | |  | 1.45 | | 0.99 to 2.12 | | | 0.057 | 0.063 | | |
| Diagnosis duration | 1.04 | 0.99 to 1.10 | 0.157 | | 0.247 | |  | 1.07 | | 1.01 to 1.13 | | | 0.019 | 0.042 | | |
| BMI | 0.92 | 0.84 to 1.00 | 0.054 | | 0.099 | |  | 0.88 | | 0.80 to 0.97 | | | 0.010 | 0.030 | | |
| Night pain | 0.96 | 0.83 to 1.11 | 0.598 | | 0.662 | |  | 1.16 | | 1.00 to 1.34 | | | 0.048 | 0.059 | | |
| PGA | 1.03 | 0.90 to 1.19 | 0.662 | | 0.662 | |  | 1.16 | | 1.01 to 1.35 | | | 0.041 | 0.056 | | |
| Sacroiliitis average | 2.08 | 1.44 to 3.02 | 0.001 | | 0.006 | |  | 1.90 | | 1.31 to 2.75 | | | 0.001 | 0.005 | | |
| Hip involvement | 2.08 | 1.55 to 5.20 | 0.001 | | 0.006 | |  | 2.90 | | 1.55 to 5.43 | | | 0.001 | 0.005 | | |

BASMI, Bath Ankylosing Spondylitis Metrology Index; BASFI, Bath Ankylosing Spondylitis Functional Index;

BMI, body boss index; PGA, patient global assessment. FDR, False Discovery Rate. OR, odds ratio; 95% CI,

95% confidence interval. Sacroiliitis average, means average radiological grade of the sacroiliac joint.
