# Supplementary material for: Interaction effects of significant risk factors on low bone mineral density in ankylosing spondylitis
Source: PeerJ. 2023 Nov 22;11:e16448. doi: 10.7717/peerj.16448 (PMC10676083; doi:10.7717/peerj.16448)
Supplement: Supplemental Information 5 — AIC, Akaike information criterion; BIC, Bayesian information criterion; LR, likelihood ratio; NRI, net reclassification improvement; IDI, integrated discrimination improvement; AUROC, area under the receiver operating characteristic; Ref., reference. [file peerj-11-16448-s005.docx]

**Table S5:**

**Prediction accuracy gained by adding the identified significant factors for low BMD in validation set.**

| **Statistic** | **Femoral neck** | | | **Total hip** | | |
| --- | --- | --- | --- | --- | --- | --- |
|  | **Basic model** | | **Full model** | **Basic model** | | **Full model** |
| **Calibration** |  | |  |  | |  |
| **AIC** | 188 | | 174 | 183 | | 187 |
| **BIC** | 268 | | 271 | 280 | | 255 |
| **LR test (χ^2^)** | Ref. | | 25.33 | Ref. | | 26.19 |
| **LR test (*P* value)** | Ref. | | <0.001 | Ref. | | <0.001 |
| **Discrimination** |  | |  |  | |  |
| **NRI (*P* value)** | Ref. | | 0.002 | Ref. | | 0.050 |
| **IDI (*P* value)** | Ref. | | <0.001 | Ref. | | <0.001 |
| **AUROC** | 0.671 | 0.788 | | 0.672 | 0.799 | |
| **AUROC (*P* value)** | 0.031 | | | <0.001 | | |

AIC, Akaike information criterion; BIC, Bayesian information criterion; LR, likelihood ratio; NRI, net reclassification improvement; IDI, integrated discrimination improvement; AUROC, area under the receiver operating characteristic; Ref., reference.
