# Supplementary material for: Interaction effects of significant risk factors on low bone mineral density in ankylosing spondylitis
Source: PeerJ. 2023 Nov 22;11:e16448. doi: 10.7717/peerj.16448 (PMC10676083; doi:10.7717/peerj.16448)
Supplement: Supplemental Information 7 [file peerj-11-16448-s007.docx]

| abbreviation | annotation |
| --- | --- |
| lbmd | bone mineral density of lumbar spine |
| lt | T-scores of lumbar spine |
| lz | Z-scores of lumbar spine |
| fnbmd | bone mineral density of femoral neck |
| fnt | T-scores of femoral neck |
| fz | Z-scores of lumbar spine |
| hipbmd | bone mineral density of total hip |
| hipt | T-scores of total hip |
| hipz | Z-scores of total hip |

Codebook of raw data

| abbreviation | annotation |
| --- | --- |
| lbmd | bone mineral density of lumbar spine |
| lt | T-scores of lumbar spine |
| lz | Z-scores of lumbar spine |
| fnbmd | bone mineral density of femoral neck |
| fnt | T-scores of femoral neck |
| fz | Z-scores of lumbar spine |
| hipbmd | bone mineral density of total hip |
| hipt | T-scores of total hip |
| hipz | Z-scores of total hip |

Notes for the second categorical variable

| categorical variable | annotation |
| --- | --- |
| gender | 0 represents female, 1 represents male. |
| sport | 0 represents no exercise, 1 represents exercise |
| alcohol_history | 0 represents no history of alcohol, 1 represents a history of alcohol. |
| alcohol_frequency | 0 represents infrequent drinking and 1 represents frequent drinking. |
| dalliy_alcohol | 0 represents no daily alcohol consumption, 1 represents daily alcohol consumption. |
| current_tnf_i | 0 represents no current use of TNF-α inhibitors, 1 represents current use of TNF-α inhibitors. |
| current_cdmards | 0 represents no use of DMARDs currently, and 1 represents current use of DMARDs. |
| current_nsaids | 0 represents no current use of NSAIDs, 1 represents current use of NSAIDs. |
| family_history | 0 indicates no family history, 1 indicates family history. |
| ever_smoking | 0 represents no smoking history, 1 represents smoking history. |
| current_smoking | 0 represents non-smoker, 1 represents current smoker. |
| hla_b27 | 0 represents HLA-B27 negative, 1 represents HLA-B27 positive. |
| hipinvolvement | 0 represents no involvement of hip joint, and 1 represents involvement of hip joint. |
