# Supplementary material for: Interaction effects of significant risk factors on low bone mineral density in ankylosing spondylitis
Source: PeerJ. 2023 Nov 22;11:e16448. doi: 10.7717/peerj.16448 (PMC10676083; doi:10.7717/peerj.16448)
Supplement: Supplemental Information 8 [file peerj-11-16448-s008.zip › Case Report Form( original).pdf]

患者姓名缩写	编号

中日友好医院课题

强直性脊柱炎患者放射学特征及进展影响因素和中药干预作用的长期队列研究

病例报告表初查

(Case Report Form)

队列研究
版本号：20170702

时间：______________________

患者姓名缩写：________________

研究者签名：__________________

研究负责人签名：______________

卫生部中日友好医院

填写说明

1.本表格请用蓝黑色或黑色钢笔或签字笔填写，不得用铅笔填写。

2.筛选合格者填写本表。

3.需要选择的项目前面有□，请在□上划“✓”即可。

4.表格中各项内容应认真、如实填写，不得随意涂改。如果填写错误，更改处不能涂黑，只能在更改处居中划一横线，并签署修改者姓名和修改时间。

5.受试者姓名拼音缩写四格需填满，两字姓名填写拼音前两个字母；三字姓名填写三字首字母及第三字第二个字母；四字姓名填写每一个字的第一个字母，如：王涛WATA，刘淑英LSYI；欧阳绿萍OYLP。

6.实验室检查表格中“检查日期”一栏按照化验单上打印的检验日期填写。检查值一项直接填写本研究中心化验报告单上的结果即可，不需注明单位。

7.所有检验项目因故未查或漏查，请填写 ND；具体用药剂量和时间不明，请填写NK。

8.应如实填写不良事件记录表，如有严重不良事件发生，请及时上报本中心研究负责人。

研究负责单位：卫生部中日友好医院

联系人：孔维萍

电话：010－84205067

传真：010－84205152

患者姓名拼音字母	就诊日期	病例筛选
□□□□	|_|_|_|_|/|_|_|/|_|_| 年　月　日	

表 1　一般情况

姓名			受试者姓名首字母	___ ___ ___ ___		
年龄	岁		性别	□ 男　□ 女		
民族			出生日期	年　月　日		
发病年龄	岁	病程　月	AS 家族史	□ 是 □ 否		
发病形式	□急性　□慢性					
首发部位						
□门诊 □ 住院（病案号　）			婚姻情况	□已婚 □ 未婚 □离异 □ 丧偶		
HLA-B27：　□阴性　□阳性						

表 2　1984 年修订的强直性脊柱炎纽约标准

临床标准	①腰痛、晨僵 3 个月以上，活动改善，休息无改善；②腰椎额状面和矢状面活动受限；③胸廓活动度低于相应年龄、性别正常人。
放射学标准	骶髂关节炎分级见上：双侧≥II 级或单侧III—IV级骶髂关节炎。
诊　断	肯定 AS：符合放射学标准和 1 项（及以上）临床标准者
符合诊断标准　□ 是 □ 否	

表 3　2009 修订的中轴型 SPA 分类标准、2011 年修订的外周型 SPA 分类标准

2009 年修订的中轴型 SpA 分类标准	
临床标准	腰背痛≥3 个月且发病年龄小于 45 岁的患者
SpA 特征	1.炎性腰背痛 2.关节炎 3.肌腱附着点炎 4.葡萄膜炎 5.指（趾）炎 6.银屑病 7.克罗恩病 / 溃疡性结肠炎 8.NSAID 治疗有效 9.具有 SpA 家族史 10.HLA-B27 阳性 11.CRP 升高
诊断标准	影像学现实骶髂关节炎且具有≥1 个 SpA 特征或 HLA-B27 阳性且具有≥2 个 SpA 特征
是否符合诊断标准	□ 是 □ 否
2011 年修订的外周型 SPA 分类标准	
临床标准	关节炎或附着点炎或指（趾）炎
SpA 特征 1	1.银屑病 2.炎性肠病 3.前驱感染 4.HLA-B27 阳性 5.葡萄膜炎 6.骶髂关节炎影像学证据（X 线或 MRI）
SpA 特征 2	1.关节炎 2.起止点炎 3. 指（趾）炎 4.IBP 既往史 5.SpA 家族史
诊断标准	临床标准加上≥1 条 SpA 特征 1 或加上≥2 条 SpA 特征 2
是否符合诊断标准	□ 是 □ 否

表 4 入选标准、排除标准

入选标准	是	否
1 符合美国风湿学会 1984 年修订的强直性脊柱炎纽约标准；（见上表）	□	□
2 符合 2009 年 ASAS 的中轴型 SpA 的分类标准，或符合 2011 年 ASAS 的外周型 SpA 分类标准。		
3 符合中医证候诊断标准；（见上表）	□	□
4 年龄＞16岁者；	□	□
5 自愿作为受试对象，签署知情同意书；	□	□
如以上任何一个答案为“否”，此受试者不能参加试验		
排除标准	是	否
1 不符合上述纳入标准者；	□	□
2 妊娠或哺乳期妇女及精神病患者，严重感染者，合并重度营养不良，或伴有心、脑、肾、造血系统严重损害		
如以上任何一个答案为“是”，此受试者不能参加试验		

筛查合格：□ 是 □ 否
是否签署知情同意书：□ 是 □ 否
入组日期：__ __ __ __ 年 __ __ 月__ __ 日

研究者签名：__________

患者姓名拼音字母<br><br>□□□□	就诊日期<br>|_|_|_|_|/|_|_|/|_|_| 年 月 日	访视一<br>0 周

表 1 背景信息

1、您的髋部或背部疼痛出现的时间？

__ __/__ __/__ __ __ __

2、诊断强直性脊柱炎的时间。

__ __/__ __/__ __ __ __

3. 您是否曾使用过以下药物：

药品名称			
柳氮磺吡啶	是	否	不知道
甲氨蝶呤	是	否	不知道
硫唑嘌呤	是	否	不知道
麦考酚酸酯	是	否	不知道
羟氯喹	是	否	不知道
强的松	是	否	不知道
唑来膦酸	是	否	不知道
伊班膦酸	是	否	不知道
阿仑膦酸盐	是	否	不知道
利塞膦酸盐	是	否	不知道

4.您是否使用过以下生物制剂？

				开始时间	停止时间
英夫利昔单抗	是	否	不知道	________	_______
依那西普	是	否	不知道	________	_______
阿达木单抗	是	否	不知道	________	_______
戈利木单抗	是	否	不知道	________	_______
赛妥珠单抗	是	否	不知道	________	_______
其他生物制剂	是	否	不知道	________	_______
益赛普	是	否	不知道	________	_______
强克	是	否	不知道	________	_______

5a：您是否吸烟超过 100 支？是　否　不知道

5b：您开始吸烟的时间？__________________　您戒烟的时间？________

6、您现在是否吸烟？是　每天多少支？　否　您以前每天吸多少支？

7. 您有下面的病症吗？

病症	有	无	不知道	最早出现症状的年份及月份	诊断的年份及月份
虹膜炎/葡萄膜炎					
银屑癣					
反应性关节炎/莱特尔综合征					
溃疡性结肠炎					
克罗丁					
跟腱炎/足底筋膜炎					
关节炎*					
背部僵直疼痛					
跟痛症					

*　除去腰椎和髋关节

如果有该病症，请填写其最早出现症状及诊断的年份、月份。

表 3 测量表

身高：　　　厘米　　　　体重：　　　千克

度量 BIM 公式:　　　　　　Kg/cm^2（千克/厘米^2）

腰围：　　　厘米　　　　血压：　　/　　　心率：　　次/分

髋关节	髋关节内旋	髋关节外旋	总髋关节旋转度
右	° ＋	° ＝	°
左	° ＋	° ＝	°

颈椎	右	左	总颈椎旋转度
颈椎旋转	° ＋	° ＝	°
颈椎侧弯	° ＋	° ＝	°

侧腰弯曲	直立时指尖到地面的距离	弯腰时指尖到地面的距离	总侧腰弯曲度
右	cm －	cm ＝	cm
左	cm －	cm ＝	cm

枕墙距　　　　cm	胸廓活动度　　　　cm

	弯腰	直立	Schober 评分
Schober 试验	cm －	10 cm ＝	cm

最大踝间距	

肌腱端指数测量表

	疼痛	
	无	有
C1-2		
C7-T1		
T12-L1		
L5-S1		

	右		左	
	疼痛		疼痛	
	无	有	无	有
大转子				
膝关节内收				
髂前上棘				
坐骨结节				
跟腱插入				
足底插入				

总肌腱端评分：

关节肿胀疼痛指数

关节<br><br>（圈出合适的答案）	右					左			
	疼痛		肿胀			疼痛		肿胀	
	无	有	无	有		无	有	无	有
胸锁关节	0	1	0	1		0	1	0	1
肩锁关节	0	1	0	1		0	1	0	1
肩关节	0	1	0	1		0	1	0	1
肘关节	0	1	0	1		0	1	0	1
腕关节	0	1	0	1		0	1	0	1
第一掌指关节	0	1	0	1		0	1	0	1
第二掌指关节	0	1	0	1		0	1	0	1
第三掌指关节	0	1	0	1		0	1	0	1
第四掌指关节	0	1	0	1		0	1	0	1
第五掌指关节	0	1	0	1		0	1	0	1
拇指指间关节	0	1	0	1		0	1	0	1

第二指间关节	0	1	0	1		0	1	0	1
第三指间关节	0	1	0	1		0	1	0	1
第四指间关节	0	1	0	1		0	1	0	1
第五指间关节	0	1	0	1		0	1	0	1
膝关节	0	1	0	1		0	1	0	1
踝关节	0	1	0	1		0	1	0	1
第一跖趾关节	0	1	0	1		0	1	0	1
第二跖趾关节	0	1	0	1		0	1	0	1
第三跖趾关节	0	1	0	1		0	1	0	1
第四跖趾关节	0	1	0	1		0	1	0	1
第五跖趾关节	0	1	0	1		0	1	0	1
髋关节	0	1	0	0		0	1	0	0

MD Global Assessment of Arthritis Activity

不活跃 ---非常活跃
0 100

表 4 关节活动情况问卷（AAQ）

疼痛数字评分量表（NRS）

请您根据过去一周关节的活动情况打分，分值从 0 到 10 对应完成该项活动由易到难

过去一周关节活动项目	分值
1.可以不用帮助自己穿上袜子或系鞋带	
2.可以不用帮助弯腰拾起跟前掉落的笔。	
3.可以不用帮助摸到柜子的顶部	
4.可以不用帮助也不用手从椅子上坐着站起。	
5.不需要借助背的力量平躺着站起来	
6.不借助外界支撑站立 10 分钟而无不适	
7.不借助拐杖等助走工具爬 12-15 级台阶	
8.转头看肩不需要转动身体	
9.经常做家务	
10.工作或者做家务能坚持一天而无不适	

疼痛情况

分值由 0 到 10 代表疼痛程度由零到严重

	分值
1.过去一周，强直性脊柱炎给你带来的疼痛程度	
2.过去一周，夜间脊柱疼痛的程度	

自我感觉

请根据过去一周的情况，在相应的位置填写

	很少或几乎不<br>（＜1 天）	有点或有些时候<br>（1-2 天）	经常或很多时候<br>（3-4 天）	总是<br>（5-7 天）
1.我会为以前不困扰的事情而烦恼				
2.不想吃东西，食欲差				
3.负面情绪无法排解				
4.感觉自己和其他人没什么不一样				
5.不能集中注意力				
6.感觉压抑，抑郁				

7.做什么事都很费力				
8.对未来充满希望				
9.感觉自己的生活一塌糊涂				
10.感到害怕				
11.晚上睡不安稳				
12.感到快乐				
13.说话比平时要少				
14.感到孤独				
15.感觉别人不友善				
16.很享受生活				

17.有时会想哭				
18.感到悲伤				
19.感觉别人不喜欢自己				
20.没有前进的动力				

脊柱活动

请根据上一周脊柱活动情况打分，分值从 0 到 10 代表脊柱活动从严重受限到不受限。

严重受限	0	1	2	3	4	5	6	7	8	9	10	不受限

其他情况

请根据过去的一周情况打分，分值从 0 到 10 代表程度从无到严重，

其他情况	分值
1.请为你所感到的疲劳程度评分	
2.请为你感到的颈部，背部和髋部的疼痛评分	
3.请为你的关节疼痛或肿胀程度评分（除去颈部，背部和髋部的关节）	
4.请为其他部位的触痛和压痛评分	
5.请为晨僵的程度评分	
6.晨僵持续时间（分值从 0 到 10 代表从无到 2h 及 2h 以上）	

表 5 其他疾病情况

除了强直性脊柱炎外，医生是否告知您同时患有其他疾病？

心血管疾病

冠心病	☐有	☐无	☐不清楚
心力衰竭	☐有	☐无	☐不清楚
充血性心力衰竭	☐有	☐无	☐不清楚
胸痛	☐有	☐无	☐不清楚
高血压	☐有	☐无	☐不清楚
房颤	☐有	☐无	☐不清楚
传导阻滞或心律失常	☐有	☐无	☐不清楚
心脏瓣膜疾病	☐有	☐无	☐不清楚

如果有，请问是哪片瓣膜出现问题？_________

心脏瓣膜置换	☐有	☐无	☐不清楚

如果有，请问置换了哪片瓣膜？__________

心脏搭桥手术	☐有	☐无	☐不清楚
心脏动脉支架	☐有	☐无	☐不清楚
中风或脑血管疾病或短暂性脑缺血	☐有	☐无	☐不清楚

其他 ____

呼吸系统疾病

肺气肿	☐有	☐无	☐不清楚
慢性支气管炎	☐有	☐无	☐不清楚
哮喘	☐有	☐无	☐不清楚
睡眠呼吸暂停	☐有	☐无	☐不清楚

其他____

恶性肿瘤

淋巴瘤	☐有	☐无	☐不清楚
白血病	☐有	☐无	☐不清楚
黑色素瘤	☐有	☐无	☐不清楚
基底细胞癌	☐有	☐无	☐不清楚
鳞状细胞癌	☐有	☐无	☐不清楚

其他____

骨科

骨折	☐有	☐无	☐不清楚

如果有，请填写骨折原因: ______ 骨折时间:__________

骨折部位:______ 是否伴有外伤:______

骨质疏松	☐有	☐无	☐不清楚
截肢	☐有	☐无	☐不清楚

其他____

胃肠道疾病

胃溃疡 □有 □无 □不清楚
憩室炎 □有 □无 □不清楚
胆囊疾病 □有 □无 □不清楚
肝脏疾病或肝硬化 □有 □无 □不清楚
痢疾 □有 □无 □不清楚
便秘 □有 □无 □不清楚
鼓胀 □有 □无 □不清楚
食管返流 □有 □无 □不清楚
其他____

神经系统疾病

帕金森病 □有 □无 □不清楚
阿兹海默病 □有 □无 □不清楚
半身不遂 □有 □无 □不清楚
脊髓受损 □有 □无 □不清楚
马尾神经综合征 □有 □无 □不清楚
外周神经病变 □有 □无 □不清楚
头痛或偏头痛 □有 □无 □不清楚
其他____

其他疾病

抑郁 □有 □无 □不清楚
糖尿病 □有 □无 □不清楚
青光眼 □有 □无 □不清楚
白内障 □有 □无 □不清楚
淀粉样变性 □有 □无 □不清楚
肾结石 □有 □无 □不清楚
IgA 肾病 □有 □无 □不清楚
超重或肥胖症 □有 □无 □不清楚
器官移植 □有 □无 □不清楚
如果有，请填写移植时间:______ 移植器官:______ 移植原因:______
其他____

表 6 药物使用情况登记表

在过去的 6 个月内，您是否服用药物？ ☐ 是 ☐ 否

请将您服用的所有药物列出来（所有的处方和非处方药）

口服药物如：抗炎药物如阿司匹林，萘普生，扶他林，布洛芬或吡罗昔康及中药、中成药等

注射或静脉滴注的药物如：益赛普，伐利木单抗，英利昔单抗或赛妥珠单抗

药物名称（请写通用名称）	每片/每次注射/每次点滴多少毫克（毫升）	过去六个月内服药几个月	上月服药多少天（片剂）	上周服药多少天（片剂）	每日服用量（片剂）	每周注射量（注射或滴注）	每个月注射量（注射或滴注）	是否仍在服用

表 7 检验检查(见下页)

检查日期	检查项目	具体值		是否异常	是否有临床意义
__年__月__日	血沉（ESR）			□是 □否	□是 □否
__年__月__日	C-反应蛋白			□是 □否	□是 □否
__年__月__日	血常规	白细胞		□是 □否	□是 □否
		红细胞		□是 □否	□是 □否
		血红蛋白		□是 □否	□是 □否
		血小板		□是 □否	□是 □否
__年__月__日	肝功能	ALT		□是 □否	□是 □否
		AST		□是 □否	□是 □否
		总胆红素		□是 □否	□是 □否
		直接胆红素		□是 □否	□是 □否
__年__月__日	肾功能	肌酐		□是 □否	□是 □否
		尿素氮		□是 □否	□是 □否
__年__月__日	骨密度	腰椎：BMD:_____ T 值：_____ Z 值：__<br>股骨颈：BMD:_____ T 值：_____ Z 值：__<br>全髋：BMD:_____ T 值：_____ Z 值：__			
__年__月__日	颈椎 X 线片（正侧位）	评分：			
__年__月__日	腰椎 X 线片（正侧位）	评分：			
__年__月__日	骶髂关节 X 线片	左侧分级：		右侧分级	
__年__月__日	骶髂关节 CT	左侧分级：		右侧分级	
__年__月__日	骶髂关节 MRI	左侧活动性炎症：		右侧活动性炎症：	
__年__月__日	双髋 X 线	左侧分级：		右侧分级：	
__年__月__日	双髋 CT	左侧分级：		右侧分级：	

患者姓名拼音字母	就诊日期	
□□□□	|_|_|_|_|/|_|_|/|_|_| 年 月 日	不良事件

检查单粘贴页面
