# Supplementary material for: Interaction effects of significant risk factors on low bone mineral density in ankylosing spondylitis
Source: PeerJ. 2023 Nov 22;11:e16448. doi: 10.7717/peerj.16448 (PMC10676083; doi:10.7717/peerj.16448)
Supplement: Supplemental Information 8 [file peerj-11-16448-s008.zip › Case Report Form(English translation).docx]

| **Patient's initials** | **Number** |
| --- | --- |

**China-Japan Friendship Hospital Project**

**Long term cohort study on the radiological characteristics and progress of patients with ankylosing spondylitis**

Preliminary investigation of case report form

**(Case Report Form)**

**Cohort study**

**Version number:** **20170702**

Time:

Patient's initials:

Researcher's signature:

Signature of the research leader:

China Japan Friendship Hospital

**Fill in instructions**

1. Please fill out this form with a blue black or black pen or signature pen, and do not use a pencil.

2. Fill out this form for those who pass the screening process.

3. The items that need to be selected are preceded by □, please mark "Å" on the □.

4. All contents in the form should be filled out carefully and truthfully, and cannot be arbitrarily altered. If there is an error in filling out, the changes cannot be blacked out, only a horizontal line can be drawn in the center of the changes, and the name of the modifier and the modification time can be signed.

5. The four pinyin abbreviations of the subject's name must be filled in, and the first two letters of the pinyin should be filled in for the two character name; Fill in the first letter and second letter of the three character name; Fill in the first letter of each character with a four character name, such as: Wang Tao WATA, Liu Shuying LSYI; Ouyang Lvping OYLP.

6. Fill in the "Inspection Date" column in the laboratory inspection form according to the inspection date printed on the test form. The inspection value can be directly filled in the results on the laboratory report form of our research center, without specifying the unit.

7. If all inspection items were not inspected or missed due to reasons, please fill in the ND; The specific dosage and time of medication are unknown, please fill in NK.

8. The adverse event record form should be truthfully filled out. If there are serious adverse events, please report them to the research leader of our center in a timely manner.

Research responsible unit: China Japan Friendship Hospital Contact person: Kong Weiping

Tel: 010-84205067

Fax: 010-84205152

| Patient name pinyin letters | Visit Date | Case screening |
| --- | --- | --- |
| □□□□ | \|_\|_\|_\|_\|/\|_\|_\|/\|_\|_\|  specific date |  |

**Table** **1** **General situation**

| full name |  | | Initial of subject's name | | ___ ___ ___ ___ | |
| --- | --- | --- | --- | --- | --- | --- |
| Age |  | | Gender | | □ Male □ Female | |
| nation |  | | date of birth | specific date | | |
| Age of onset |  | Disease duration month | | AS family history | | □ Yes □ No |
| Pathogenic form | □ Acute □ Chronic | | | | | |
| Initial location |  | | | | | |
| □ Outpatient □ Inpatient (medical record number) | | | Marital situation | □ Married □ Unmarried □ Divorced □ Widowed | | |
| HLA-B27: □ Negative □ Positive | | | | | | |

**Table 2 Revised New York Standard for Ankylosing Spondylitis in 1984**

| Clinical standards | ① Low back pain, morning stiffness for more than 3 months, improved activity, no improvement in rest; ② Limited activity in frontal and sagittal plane planes of lumbar spine; ③ The chest mobility is lower than that of normal individuals of the corresponding age and gender. |
| --- | --- |
| Radiological standards | The classification of sacroiliac arthritis is as follows: bilateral ≥ grade II or unilateral grade III-IV sacroiliac arthritis. |
| diagnosis | Positive AS: Those who meet radiological standards and one (or more) clinical standard |
| Meets diagnostic criteria □ Yes □ No | |

**Table** **3: 2009 Revised Central Axis SPA Classification Standard and 2011 Revised Peripheral SpA Classification Standard**

| **Revised** **classification standard for axial** **SpA in 2009** | |
| --- | --- |
| Clinical standards | Patients with low back pain ≥ 3 months old and onset age less than 45 years old |
| SpA characteristics | 1. Inflammatory low back pain 2. Arthritis 3. Tendon attachment point inflammation 4. Uveitis 5. Finger (toe) inflammation 6. Psoriasis 7. Krohn's disease/ulcerative colitis 8. NSAID treatment effective 9. SpA family history 10. HLA-B27 positive 11. CRP increase |
| diagnostic criteria | Imaging reality of sacroiliac arthritis with ≥ 1 SpA feature or HLA-B27 positive with ≥ 2 SpA features |
| Does it meet diagnostic criteria | □ Yes □ No |
| **Revised** **classification standard for peripheral** **SPA in 2011** | |
| Clinical standards | Arthritis or attachment point inflammation or finger (toe) inflammation |
| SpA feature 1 | 1. Psoriasis 2. Inflammatory bowel disease 3. Precursor infection 4. HLA-B27 positive 5. Uveitis 6. Imaging evidence of sacroiliac arthritis (X-ray or MRI) |
| SpA feature 2 | 1. Arthritis 2. Starting and ending point inflammation 3 Finger (toe) inflammation 4. Past history of IBP 5. SpA family history |
| diagnostic criteria | Clinical standard plus ≥ 1 SpA feature 1 or plus ≥ 2 SpA features 2 |
| Does it meet diagnostic criteria | □ Yes □ No |

**Table** **4 Inclusion and Exclusion Criteria**

| Inclusion Criteria | yes | no |
| --- | --- | --- |
| 1. Comply with the New York Standard for Ankylosing Spondylitis revised by the American Rheumatology Society in 1984; (see table above) | □ | □ |
| 2. Comply with the classification standards of the 2009 ASAS axial type SpA or the 2011 ASAS peripheral type SpA. |  |  |
| 3. Meet the diagnostic criteria for traditional Chinese medicine syndromes; (see table above) | □ | □ |
| 4. Those over 16 years old; | □ | □ |
| 5. Volunteer as the subject and sign an informed consent form; | □ | □ |
| If any of the above answers are 'no', this subject cannot participate in the experiment | | |
| Exclusion criteria | yes | no |
| 1. Those who do not meet the inclusion criteria mentioned above; | □ | □ |
| Pregnant or lactating women and psychiatric patients, severely infected individuals, combined with severe malnutrition, or accompanied by severe damage to the heart, brain, kidneys, and hematopoietic system |  |  |
| If any of the above answers are 'yes', this subject cannot participate in the experiment | | |

**Screening qualified**: □ Yes □ No

**Whether** to **sign the informed consent form**: □ Yes □ No

**Joined on**: __ __ ____ Year ____ Month ____ day

**Researcher's signature:__________**

| Patient name pinyin letters | Visit Date | Visit 1  0 weeks |
| --- | --- | --- |
| □□□□ | \|_\|_\|_\|_\|/\|_\|_\|/\|_\|_\|  specific date |  |

**Table** **1** **Background Information**

**1. When does your hip or back pain occur? / /**

**2. The time for diagnosing ankylosing spondylitis. / /**

**3. Have you ever used the following drugs:**

**Drug name**

**Sulfasalazine Yes No Unknown**

**Methotrexate Yes No Unknown**

**Azathioprine Yes No Unknown**

**Mycophenolate Yes No Unknown**

**Hydroxychloroquine Yes No Unknown**

**Zoledronic Yes No Unknown**

**Ibandronic Yes No Unknown**

**Aalendronic Yes No Unknown**

**4. Have you ever used the following biological agents?**

**Start Time Stop Time**

**Infliximab Yes No Unknown ________ __________**

**Inazep Yes No Unknown ________ __________**

**Adalimumab Yes No Unknown ________ __________**

**Golimumab Yes No Unknown ________ __________**

**Saituozumab** **Yes No Unknown ________ __________**

Do you not know

Do you not know

**5a: Do you smoke more than** **100 cigarettes?** **Yes No Unknown**

**5b: When did you start smoking?** _____**When did you quit smoking?** _________

**6. Do you smoke now?**

**Yes**  **How many pieces per day? ______**

**No How many cigarettes did you smoke every day before? _____**

**7. Do** **you have** **the** **following symptoms?**

| **disease** | **Yes** | **NO** | **I don't know** | **The year and month when the symptoms first appeared** | **Year and month of diagnosis** |
| --- | --- | --- | --- | --- | --- |
| Iritis/uveitis |  |  |  |  |  |
| psoriasis |  |  |  |  |  |
| Reactive arthritis/Laiter syndrome |  |  |  |  |  |
| Ulcerative colitis |  |  |  |  |  |
| Crodine |  |  |  |  |  |
| Achilles tendinitis/plant fasciitis |  |  |  |  |  |
| Arthritis* |  |  |  |  |  |
| Back stiffness and pain |  |  |  |  |  |
| Heel pain syndrome |  |  |  |  |  |

* If you have this condition, please fill in the earliest symptoms and the year and month of diagnosis.

**Table** **3** **Measurement Table**

**Height: ___ cm Weight: __kg**

**Measurement** **BIM** **formula: ____Kg/cm** **²**

**Waist circumference: ___cm Blood pressure: ___/__ Heart rate:__times/minute**

| **hip joint** | **Hip joint internal rotation** | | | **External rotation of hip joint** | | | | **Total hip joint rotation** | |
| --- | --- | --- | --- | --- | --- | --- | --- | --- | --- |
| **right** | **°** **＋** **°** **＝** **°** | | | | | | | | |
| **Left** | **°** **＋** **°** **＝** **°** | | | | | | | | |
| **cervical vertebra** | **right** | | | | **Left** | | | | **Total cervical rotation** |
| **Cervical rotation** | **°** **＋** **°** **＝** **°**  **°** **＋** **°** **＝** **°** | | | | | | | | |
| **Cervical scoliosis** |  |  |  |  |  |  |  |  |  |
| **Lateral lumbar curvature** | | **Distance from fingertip to ground when standing upright** | | | | **The distance between the fingertips and the ground when bending down** | | | **Total lateral lumbar curvature** |
| **right** | | **cm** **－** **cm** **＝** **cm**  **cm** **－** **cm** **＝** **cm** | | | | | | | |
| **Left** | |  |  |  |  |  |  |  |  |
| **Sleeper wall distance** **cm** | | | | | | | **Chest mobility** **cm** | | |
|  | | | **bend** | | | | **erect** | | **Schober** **rating** |
| **Schober** **test** | | | **cm** **－** **10 cm** **＝** **cm** | | | | | | |
| **Maximum ankle spacing** | | |  | | | | | | |

**Tendon end index measurement table**

|  | **pain** | | | | |
| --- | --- | --- | --- | --- | --- |
|  | **No** | | **Yes** | | |
| **C1-2** |  | |  | | |
| **C7-T1** |  | |  | | |
| **T12-L1** |  | |  | | |
| **L5-S1** |  | |  | | |
|  | **right** | | | **Left** | |
|  | **pain** | | | **pain** | |
|  | **No** | **Yes** | | **No** | **Yes** |
| **Large rotor** |  |  | |  |  |
| **Knee joint adduction** |  |  | |  |  |
| **Anterior superior iliac spine** |  |  | |  |  |
| **Ischial tuberosity** |  |  | |  |  |
| **Achilles tendon insertion** |  |  | |  |  |
| **Plantar insertion** |  |  | |  |  |

| **Total tendon end score:** |
| --- |

**Joint swelling and pain index**

| **joint**  **(Circle the appropriate answer)** | **right** | | | | **Left** | | | |
| --- | --- | --- | --- | --- | --- | --- | --- | --- |
|  | **pain** | | **swelling** | | **pain** | | **swelling** | |
|  | **No** | **Yes** | **No** | **Yes** | **No** | **Yes** | **No** | **Yes** |
| **Sternoclavicular joint** | **0** | **1** | **0** | **1** | **0** | **1** | **0** | **1** |
| **Acromioclavicular joint** | **0** | **1** | **0** | **1** | **0** | **1** | **0** | **1** |
| **shoulder joint** | **0** | **1** | **0** | **1** | **0** | **1** | **0** | **1** |
| **elbow joint** | **0** | **1** | **0** | **1** | **0** | **1** | **0** | **1** |
| **wrist joints** | **0** | **1** | **0** | **1** | **0** | **1** | **0** | **1** |
| **First MCP** | **0** | **1** | **0** | **1** | **0** | **1** | **0** | **1** |
| **Second MCP** | **0** | **1** | **0** | **1** | **0** | **1** | **0** | **1** |
| **Third MCP**  **halangeal joint** | **0** | **1** | **0** | **1** | **0** | **1** | **0** | **1** |
| **Fourth MCP** | **0** | **1** | **0** | **1** | **0** | **1** | **0** | **1** |
| **Fifth MCP** | **0** | **1** | **0** | **1** | **0** | **1** | **0** | **1** |
| **PIP of thumb** | **0** | **1** | **0** | **1** | **0** | **1** | **0** | **1** |
| **Second PIP** | **0** | **1** | **0** | **1** | **0** | **1** | **0** | **1** |
| **Third PIP** | **0** | **1** | **0** | **1** | **0** | **1** | **0** | **1** |
| **Fourth PIP** | **0** | **1** | **0** | **1** | **0** | **1** | **0** | **1** |
| **Fifth PIP** | **0** | **1** | **0** | **1** | **0** | **1** | **0** | **1** |
| **Knee joint** | **0** | **1** | **0** | **1** | **0** | **1** | **0** | **1** |
| **ankle joint** | **0** | **1** | **0** | **1** | **0** | **1** | **0** | **1** |
| **first MTP** | **0** | **1** | **0** | **1** | **0** | **1** | **0** | **1** |
| **Second MTP** | **0** | **1** | **0** | **1** | **0** | **1** | **0** | **1** |
| **Third MTP** | **0** | **1** | **0** | **1** | **0** | **1** | **0** | **1** |
| **Fourth MTP** | **0** | **1** | **0** | **1** | **0** | **1** | **0** | **1** |
| **Fifth MTP** | **0** | **1** | **0** | **1** | **0** | **1** | **0** | **1** |
| **hip joint** | **0** | **1** | **0** | **0** | **0** | **1** | **0** | **0** |

**MD Global Assessment of Arthritis Activity**

**Inactive** **- - - - - - - - - - - - - - - - - - - - - - - - - - - - - - - - - - - - - - - - - - - - - - - - - - - - - -**

**Table** **4** **Joint Activity Questionnaire (AAQ)**

**Pain Digital Rating Scale (NRS)**

| **Joint activity projects in the past week** | **Score** |
| --- | --- |
| 1. **You don't need to help yourself put on socks or tie shoelaces** |  |
| 1. **You don't need to bend down to pick up the pen that fell in front of you.** |  |
| 1. **You don't need to help touch the top of the cabinet** |  |
| **4. You can sit up from a chair without help or hands.** |  |
| **5.** **Don't rely on the strength of your back to lie flat and stand up** |  |
| **6.** **Stand without external support for 10** **minutes without discomfort** |  |
| **7.** **Climbing** **12-15** **steps** **without using walking aids such as crutches** |  |
| **8.** **Turning your head to look at your shoulders does not require turning your body** |  |
| **9.** **Regularly do household chores** |  |
| **10.** **Can persist for a day without discomfort in work or household chores** |  |

**Please rate** **the joint activity based on the past week, with a score ranging from** **0** **to** **10,** **corresponding to completing the activity from easy to difficult**

**Pain situation**

**The score from 0 to 10 represents the degree of pain from zero to severe**

|  | **Score** |
| --- | --- |
| 1. **The degree of pain caused by ankylosing spondylitis in the past week** |  |
| 1. **The degree of spinal pain at night in the past week** |  |

**Self perception**

Please fill in the corresponding positions based on the situation of the past week

|  | **Very little or almost no**  **(<1** **day)** | **Sometime**  **(1-2** **days)** | **Often**  **(3-4** **days)** | **Always**  **(5-7** **days)** |
| --- | --- | --- | --- | --- |
| 1. **I will worry about things that I didn't bother with before** |  |  |  |  |
| 1. **Not wanting to eat, poor appetite** |  |  |  |  |
| 1. **Negative emotions cannot be resolved** |  |  |  |  |
| 1. **I feel like I'm no different from others** |  |  |  |  |
| 1. **Unable to concentrate** |  |  |  |  |
| 1. **Feeling depressed, depressed** |  |  |  |  |
| 1. **It takes a lot of effort to do anything** |  |  |  |  |
| 1. **Full of hope for the future** |  |  |  |  |
| 1. **Feeling like my life is in a mess** |  |  |  |  |
| 1. **Feeling scared** |  |  |  |  |
| 1. **Sleeping restlessly at night** |  |  |  |  |
| 1. **Feeling happy** |  |  |  |  |
| 1. **Speak less than usual** |  |  |  |  |
| 1. **Feeling lonely** |  |  |  |  |
| 1. **Feeling unfriendly to others** |  |  |  |  |
| 1. **Enjoy life very much** |  |  |  |  |
| 1. **Sometimes I feel like crying** |  |  |  |  |
| 1. **Feeling sad** |  |  |  |  |
| 1. **Feeling like others don't like oneself** |  |  |  |  |
| 1. **No motivation to move forward** |  |  |  |  |

**Spinal activity**

**Please rate the spinal activity based on the previous week, with a score from 0 to 10 indicating a transition from severely restricted to unrestricted spinal activity.**

| **Severely restricted** | **0** | **1** | **2** | **3** | **4** | **5** | **6** | **7** | **8** | **9** | **10** | **Unrestricted** |
| --- | --- | --- | --- | --- | --- | --- | --- | --- | --- | --- | --- | --- |

**Other situations**

**Please rate based on the situation of the past week, with a score ranging from 0 to 10 indicating a degree from zero to severe.**

| **Other situations** | **Score** |
| --- | --- |
| 1. **Please rate the level of fatigue you feel** |  |
| 1. **Please rate the pain you feel in the neck, back, and hips** |  |
| 1. **Please rate the degree of pain or swelling in your joints (excluding the joints in the neck, back, and hips)** |  |
| 1. **Please rate the tenderness and tenderness of other parts** |  |
| 1. **Please rate the degree of morning stiffness** |  |
| **6.** **Duration of morning stiffness (scores from** **0** **to** **10** **represent** **periods from zero to** **2 hours** **and** **above)** |  |

**Table** **5**  **Registration Form for Drug Use**

Have you taken medication in the past 6 months?

Please list all medications you have taken (all prescription and over-the-counter medications)

Oral drugs, such as anti-inflammatory drugs, such as aspirin, naproxen, voltarin, ibuprofen or piroxicam, traditional Chinese medicine, traditional Chinese patent medicines and simple preparations, etc

Drugs injected or intravenous drip, such as Yisaipu, Valiximab, Infliximab, or Saitouzumab

| Drug name  (Please write the common name) | How many milligrams (milliliters) per tablet/injection/drip | Taking medication for several months in the past six months | How many days (tablets) did you take medication last month | How many days (tablets) did you take medication last week | Daily dosage (tablets) | Weekly injection volume  (Injection or drip) | Monthly injection volume (injection or drip) | Are you still taking it |
| --- | --- | --- | --- | --- | --- | --- | --- | --- |
|  |  |  |  |  |  |  |  |  |
|  |  |  |  |  |  |  |  |  |
|  |  |  |  |  |  |  |  |  |
|  |  |  |  |  |  |  |  |  |
|  |  |  |  |  |  |  |  |  |
|  |  |  |  |  |  |  |  |  |
|  |  |  |  |  |  |  |  |  |
|  |  |  |  |  |  |  |  |  |
|  |  |  |  |  |  |  |  |  |

**Table** **6**  **Inspection and Inspection**

| Inspection date | Inspection items | | Specific value | | Is it abnormal | Does it have clinical significance |
| --- | --- | --- | --- | --- | --- | --- |
| __ Year__ Month__ day | Blood sedimentation rate (ESR) | |  | | □ Yes □ No | □ Yes □ No |
| __ Year__ Month__ day | C-reactive protein | |  | | □ Yes □ No | □ Yes □ No |
| __ Year__ Month__ day | routine blood test | | white blood cell |  | □ Yes □ No | □ Yes □ No |
|  |  |  | red blood cell |  | □ Yes □ No | □ Yes □ No |
|  |  |  | hemoglobin |  | □ Yes □ No | □ Yes □ No |
|  |  |  | platelet |  | □ Yes □ No | □ Yes □ No |
| __ Year__ Month__ day | liver function | | ALT |  | □ Yes □ No | □ Yes □ No |
|  |  |  | AST |  | □ Yes □ No | □ Yes □ No |
|  |  |  | total bilirubin |  | □ Yes □ No | □ Yes □ No |
|  |  |  | Direct bilirubin |  | □ Yes □ No | □ Yes □ No |
| __ Year__ Month__ day | renal function | | creatinine |  | □ Yes □ No | □ Yes □ No |
|  |  |  | Urea nitrogen |  | □ Yes □ No | □ Yes □ No |
| __Year__ Month__ day | bone density | | Lumbar spine BMD:_____ T-scores:_____ Z-scores:___  Femur neck BMD:_____ T-scores:_____ Z-scores:___  Total hip BMD:_____ T-scores:_____ Z-scores:___ | | | |
| __ Year__ Month__ day | Cervical X-ray (AP and lateral position) | | Rating: | | | |
| __ Year__ Month__ day | Lumbar X-ray (AP and lateral position) | | Rating: | | | |
| __ Year__ Month__ day | X-ray film of sacroiliac joint | | Left grading:  Right grading | | | |
| __ Year__ Month__ day | Sacroiliac joint CT | Left grading:  Right grading | | | | |
| __ Year__ Month__ day | Sacroiliac joint MRI | Left active inflammation:  Right active inflammation: | | | | |
| __ Year__ Month__ day | Double hip X-ray | Left grading:  Right grading: | | | | |
| __ Year__ Month__ day | Double hip CT | Left grading:  Right grading: | | | | |

| Patient name pinyin letters | Visit Date |  |
| --- | --- | --- |
| □□□□ | \|_\|_\|_\|_\|/\|_\|_\|/\|_\|_\|  specific date |  |

Pasting page
